# An *in vitro* study on the degradation of multispecies biofilm of periodontitis-related microorganisms by bovine trypsin

**DOI:** 10.3389/fmicb.2022.951291

**Published:** 2022-08-04

**Authors:** Jing Zhou, Xinhui Meng, Qunchao Han, Yinxue Huang, Lijun Huo, Yayan Lei

**Affiliations:** ^1^Department of Operative Dentistry, Preventive Dentistry and Endodontics, School of Stomatology, The Affiliated Stomatology Hospital, Kunming Medical University, Kunming, China; ^2^Yunnan Key Laboratory of Stomatology, Kunming, China

**Keywords:** bovine trypsin, periodontitis, multispecies biofilm, extracellular polymeric substances, biofilm degradation

## Abstract

To investigate the degradation effect of bovine trypsin on multispecies biofilm of periodontitis-related bacteria and to provide an experimental reference for exploring new methods for controlling biofilms of periodontitis-related microorganisms, the multispecies biofilm of periodontitis-related microorganisms was established. Standard strains of *Porphyromonas gingivalis, Fusobacterium nucleatum* subsp. *polymorpha*, *Actinomyces viscosus*, and *Aggregatibacter actinomycetemcomitans* were co-cultured to form the biofilm. The experimental groups were treated with bovine trypsin, distilled water was applied as the blank control group, and phosphate saline buffer (pH = 7.4) as the negative control group. Morphological observation and quantitative analysis of extracellular polymeric substances (EPS), live bacteria, and dead bacteria were conducted using a laser confocal microscope. The morphological changes of EPS and bacteria were also observed using a scanning electron microscope. The results of morphological observations of modeling were as follows. EPS aggregated as agglomerates, and bacteria flora were wrapped by them, showing a three-dimensional network structure, and channel-like structures were inside the biofilm. Live bacteria were distributed on the surface of the EPS or embedded in them, dead bacteria aggregated between live flora and the bottom layer of biofilms. After being treated with bovine trypsin, the three-dimensional network structure and the channel-like structure disappeared, and the EPS and live and dead bacteria decreased. Quantitative analysis results are as follows. When biofilm was treated for 30 s, 1 min, and 3 min, the minimum effective concentrations of bovine trypsin to reduce EPS were 2 mg/ml (*P* < 0.05), 0.5 mg/ml (*P* < 0.05), and 0.25 mg/ml (*P* < 0.05), respectively. The minimum effective concentrations of bovine trypsin to reduce the live or dead bacteria were 2 mg/ml (*P* < 0.05), 0.5 mg/ml (*P* < 0.05), and 0.5 mg/ml (*P* < 0.05), respectively. There was no significant difference in the ratio of live/dead bacteria after the biofilm was treated for 30 s with bovine trypsin at the concentration of 0.25, 0.5, 1, and 2 mg/ml (*P* > 0.05), and the minimum effective concentration to reduce the ratio of live bacteria/dead bacteria was 0.25 mg/ml (*P* < 0.05) after treatment for 1 min and 3 min. Therefore, bovine trypsin can destroy biofilm structure, disperse biofilm and bacteria flora, and reduce the EPS and bacterial biomass, which are positively correlated with the application time and concentration.

## Introduction

Dental plaque biofilm is the initiating factor of periodontitis. Periodontitis is not only a local disease of the oral cavity, but it is also closely related to diseases such as diabetes and coronary atherosclerosis, which pose a serious threat to human health (Mealey and Oates, 2006; Zijnge et al., 2010; Pérez-Losada et al., 2016; Peng et al., 2017). Biofilms, composed of microbial flora and extracellular polymeric substances (EPS), are highly ordered microbial combinations embedded in EPS (Valm, 2019), which play an important role in mediating the transition of periodontal tissues from healthy to diseased states. EPS are mainly composed of water, extracellular polysaccharides, extracellular proteins, extracellular deoxyribonucleotides (eDNA), and lipids (Yan et al., 2016). The “multi-functional network framework” formed by EPS is the basis for the expression of the biological properties of biofilms and directly determines the living environment of cells (Koo et al., 2013; Flemming et al., 2016). EPS play a role in altering microbial behavior and virulence in biofilms and can also enhance bacterial drug resistance (Gupta et al., 2016; Liu et al., 2016).

The current treatment for periodontitis mainly involves mechanically removing or controlling the biofilm on the root surface and periodontal pocket. However, due to the viscoelasticity of the biofilm, it only partially deforms rather than falls off when subjected to shear stress (Bowen et al., 2018). Mature biofilms are mechanically difficult to remove from the tooth surface, especially in hidden parts such as the adjacent root surfaces, root depressions, and root bifurcations. Antibacterial drugs such as chlorhexidine (CHX) are also used in clinical practice as a supplement to mechanical treatment methods. However, it has certain side effects, such as cytotoxicity, teeth, and fillings discoloration (Karpiński and Szkaradkiewicz, 2015), and even oral flora imbalance. Antibacterial drugs have limited lethality to bacteria in biofilms (Banar et al., 2016; Wang et al., 2020) and cannot degrade EPS (Payne et al., 2008). The presence of EPS and continued antibiotic exposure can lead to the development of genetic resistance in bacteria, resulting in the persistence of biofilm infections (Ciofu et al., 2017). Therefore, targeting EPS may be an effective breakthrough point for removing or controlling biofilms.

Biofilm inhibitors have gradually become a research hotspot. The reported biofilm inhibitors include fibrinolytics (Hogan et al., 2018), metalloproteinases (Saggu et al., 2019), antimicrobial peptides (Zhang et al., 2019), and other eradication agent (Verderosa et al., 2019). Antibiofilm formulations targeting EPS have also been reported (Singh et al., 2002). Trypsin is a serine proteolytic enzyme that affects the formation of the intercellular skeleton to disperse cells (Niazi et al., 2014) and can significantly reduce the biofilm of oral actinomycetes and inhibit the formation of biofilms (Mugita et al., 2017). Studies by Niazi et al. (2015) have shown that the simultaneous action of trypsin with 2% chlorhexidine and ultrasound can effectively reduce the total bacterial survival in a single root canal and destroy the biofilm. However, the role and influence of trypsin on periodontitis-related biofilms are still unknown.

Therefore, in this experiment, a multispecies biofilm model of periodontitis-related microorganisms was established *in vitro*. After the action of bovine trypsin, the changes in biofilm EPS, live bacteria, dead bacteria, and the live bacteria/dead bacteria ratio were detected. The influence of the bovine trypsin on periodontitis-related microbial multispecies biofilm are expected to provide an experimental reference for the new way of the removal of periodontitis-related microbial multispecies biofilm.

## Materials and methods

### Materials and equipment

*Actinomyces viscous* (ATCC19246, China Industrial Microbial Culture Collection and Management Center); *Fusobacterium nucleatum* subsp. *polymorpha* (CGMCC1.2528, China General Microorganism Culture Collection and Management Center); *Aggregatibacter actinomycetemcomitans* (NCTC9710, China Common Microbial Bacteria Species Collection Management Center); *Porphyromonas gingivalis* (ATCC33277, China Industrial Microorganism Culture Collection Management Center); Bovine trypsin (Sigma-Aldrich, MO, United States); PBS buffer (pH 7.4) (Beyotime, Shanghai, China); Dextran, Alexa Fluor™ 647 (Thermo Fisher Scientific, Waltham, MA, United States); LIVE/DEAD BacLight Bacterial Viability Kit (Thermo Fisher Scientific, Waltham, MA, United States); BacLight™ mounting oil (Thermo Fisher Scientific, Waltham, MA, United States); 6-well plate (Corning, NY, United States); Coverslip (24 mm × 24 mm) (HaiLun, Nantong, China); Brain heart leachate broth (HuanKai Microbial, Guangzhou, China); Tri-Gas INCUBATOR (Heal Force, Hong Kong, China); Confocal laser scanning electron microscope (Nikon, Tokyo, Japan); and Scanning Electron Microscope (FEI, Eindhoven, Netherlands).

### Preparation of bovine trypsin solution

100 mg Bovine trypsin was dissolved in 10 ml of PBS buffer (pH = 7.4) to form 10 mg/ml of trypsin solution, which was diluted to the required concentration according to the experimental needs, and preheated in a constant temperature water bath at 37°C for 30 min.

### Establishment of multispecies biofilms of periodontitis-related microorganisms

The bacteria were recovered in the logarithmic phase according to the growth curve of each species, the concentration of each bacteria solution was adjusted to 1 × 10^8^ CFU/ml, and the bacteria solutions were mixed in equal proportions. The pretreated coverslips (4% HF treatment for 3 min, then sterilized and dried) were put into a sterile 6-well plate. Then, 200 μl of mixed bacterial solution and 1.8 ml of fresh sterile BHI liquid medium were added (1% sucrose by mass, 1% hemin-vitamin K_1_ solution by volume, 10% fetal bovine serum by volume) and incubated aerobically at 37°C (80% N_2_, 10% CO_2_, 10% H_2_) for 2 weeks, and the medium was replaced every 2 days.

### Experimental groups

The treatment times of bovine trypsin were 30 s (group A), 1 min (group B), and 3 min (group C). The bovine trypsin concentration of each group was as follows. Group A_1_ was 0.25 mg/ml, A_2_ 0.5 mg/ml, A_3_ 1 mg/ml, and A_4_ 2 mg/ml. Group B_1_ was 0.25 mg/ml, B_2_ 0.5 mg/ml, and B_3_ 1 mg/ml. Group C_1_ was 0.125 mg/ml, C_2_ 0.25 mg/ml, C_3_ 0.5 mg/ml, and C_4_ 1 mg/ml. The blank control group was treated with distilled water, and the negative control group was treated with PBS buffer.

### Scanning electron microscope observation of periodontitis-related microbial multispecies biofilm

The slides with biofilms were placed in 2.5% glutaraldehyde solution, fixed at 4°C for 2 h, washed three times with sterile distilled water, dehydrated with alcohol gradient, dried, and sprayed with gold.

### Confocal laser scanning microscopy observation of periodontitis-related microbial multispecies biofilm

#### Observation of live and dead bacteria

In the dark, the coverslips were washed three times with sterile distilled water. Then, 200 μl of SYTO 9/PI mixed fluorescent dye solution was added to each slide, allowed to stand for 15 min under anaerobic conditions in a 37°C incubator, and washed again three times with sterile distilled water. The slides were air dried naturally, 200 μl of anti-fluorescence quencher was applied and sealed for inspection. Three areas were randomly observed on each slide. SYTO 9 excitation/emission wavelengths were 480/500 nm and PI excitation/emission wavelengths were 490/635 nm. Images were generated by 3D reconstruction of biofilms using NIS-Elements AR Analysis software. The experiment was repeated three times.

#### Observation of extracellular polymeric substances

Alexa Fluor™ 647 fluorescent dye was added during the biofilm incubation. Coverslips were washed three times with sterile distilled water in the dark. Then, 200 μl of SYTO 9 fluorescent dye was added to each slide, left for 15 min under anaerobic conditions in a 37°C incubator, and washed three times with sterile distilled water. The slides were air dried naturally, 200 μl of anti-fluorescence quencher was applied and sealed for inspection. Three areas were randomly observed on each slide. The excitation/emission wavelengths were 650/668 nm for Alexa Fluor™ 647 and 480/500 nm for SYTO 9. The biofilms were reconstructed in 3D by NIS-Elements AR Analysis software and images were generated. The experiment was repeated three times.

### Statistical analysis

Statistical analysis was performed using SPSS software version 23.0. Two independent samples *T*-test was used for comparisons between groups, while one-way ANOVA was used for comparisons within groups, measurement data were presented as mean ± SD.

## Results

### Modeling results of periodontitis-related microbial multispecies biofilm

**FIGURE 1:**
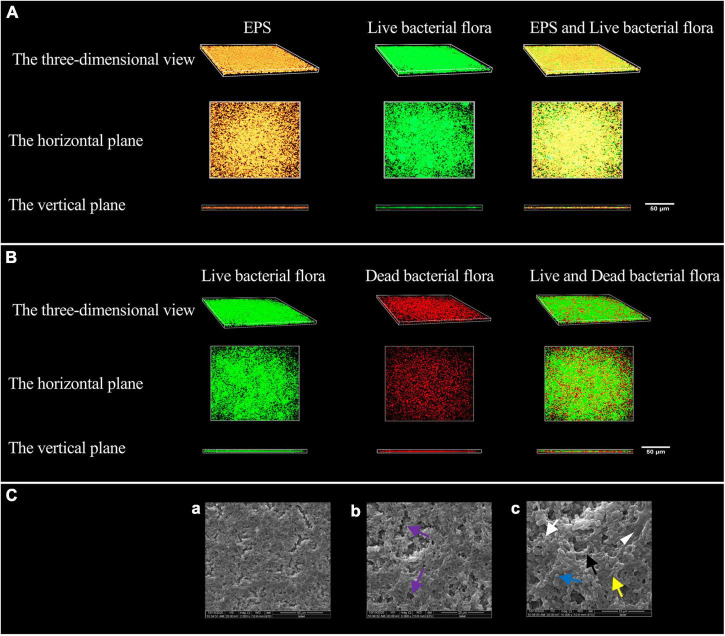
Modeling results of periodontitis-related microbial multispecies biofilm. **(A)** The three-dimensional morphology of EPS and live bacterial flora observed by CLSM. **(B)** The three-dimensional shape of the live and dead bacteria groups observed by CLSM. **(C)** The SEM observation of the biofilm morphology. **(a)** ×2000, **(b)** ×5000, and **(c)** ×10000. Channel-like structures (purple arrows) are shown in **(b)**, EPS (white triangle) in **(c)**, the short rod-shaped bacteria (white arrows) (*Actinomyces viscosus*), the paired ball-shaped bacteria (black arrows) (*Aggregatibacter actinomycetemcomitans*), the small spherical bacteria (blue arrow) (*Porphyromonas gingivalis*), and the bacteria arranged in short chains (yellow arrow) (*F. nucleatum* subsp. *polymorpha*) are shown in **(c)**.

The morphology of EPS observed by confocal laser scanning microscopy (CLSM) is shown in Figure 1A. The EPS was revealed in orange using Alexa Fluor™ 647 fluorescent dye, live bacteria were stained as green by SYTO 9. The orange aggregates are connected in the form of clumps, showing a three-dimensional network structure with micro-voids in it; the green colonies are aggregated on the surface of the orange clumps or embedded in them. The communities of live and dead bacteria are shown in Figure 1B. Dead bacteria were visualized by PI in red, live bacteria were stained as green by SYTO 9. Live bacteria gather in groups, and dead bacteria are scattered among the live bacteria groups and at the bottom of the biofilm.

The morphology of the biofilm observed by scanning electron microscope (SEM) is shown in Figure 1C. The biofilm has a three-dimensional network structure with channel-like structures of varying sizes. The biofilm contains four types of bacteria, namely short rods, pairs or clusters of rods, small globules, and short chains. The bacteria aggregate, make contact with each other, and are wrapped by EPS.

### The effect of bovine trypsin on extracellular polymeric substances in periodontitis-related microbial multispecies biofilms

#### Biofilm morphological changes after bovine trypsin treatment observed by scanning electron microscope

**FIGURE 2:**
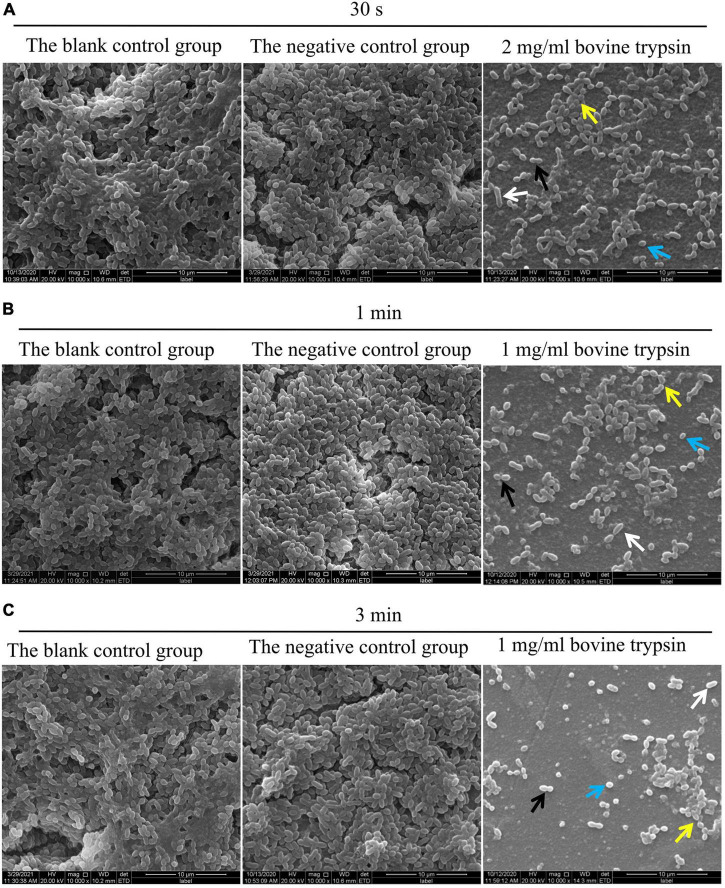
SEM observation of biofilm morphology after bovine trypsin action. **(A)** The morphology of the biofilm after 2 mg/ml bovine trypsin treatment for 30 s (×1000). **(B)** The morphology of the biofilm after 1 mg/ml bovine trypsin treatment for 1 min (×1000). **(C)** The morphology of the biofilm after 1 mg/ml bovine trypsin treatment for 3 min (×1000). The short rod-shaped bacteria (white arrows), the paired ball-shaped bacteria (black arrows), the small spherical bacteria (blue arrows), and the bacteria arranged in short chains (yellow arrows).

The SEM observation results of biofilm morphology after treatment with 2 mg/ml bovine trypsin for 30 s, 1 mg/ml bovine trypsin for 1 min, and 1 mg/ml bovine trypsin for 3 min, respectively, are shown in Figure 2. The biofilm morphology of the blank control group was similar to that of the negative control group. After bovine trypsin treatment, the biofilm became thinner, the three-dimensional network and channel-like structures disappeared, only a layer of scattered bacteria remained, and EPS were significantly reduced. However, four types of bacteria were still visible in the residual biofilm, and the bacterial morphology did not change significantly.

#### Morphological changes and quantitative analysis of extracellular polymeric substances after bovine trypsin treatment according to confocal laser scanning microscopy observation

**FIGURE 3:**
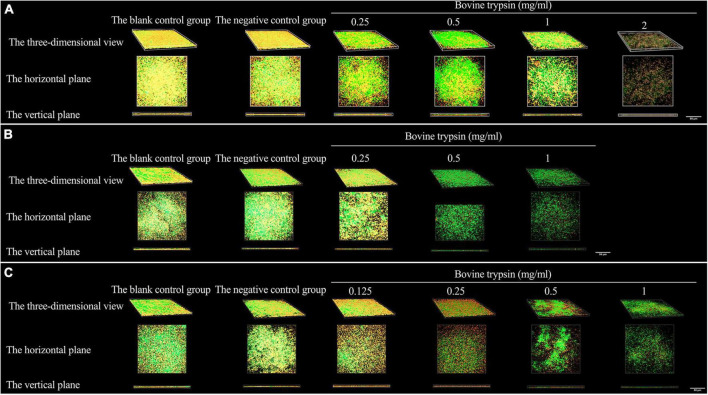
The three-dimensional morphology observation of biofilm EPS after 30 s, 1 min, and 3 min of bovine trypsin treatment by CLSM. **(A)** The morphology of EPS after 30 s of action by bovine trypsin. **(B)** The morphology of EPS after 1 min of action by bovine trypsin. **(C)** The morphology of EPS after 3 min of action by bovine trypsin.

Figure 3 shows the morphology of EPS after the biofilm was treated with bovine trypsin for 30 s, 1 min, and 3 min, respectively. With the prolongation of the action time and the increase of the action concentration, the orange fluorescence of EPS weakened and became sparse, the voids increased, and the cross-linking decreased.

**FIGURE 4:**
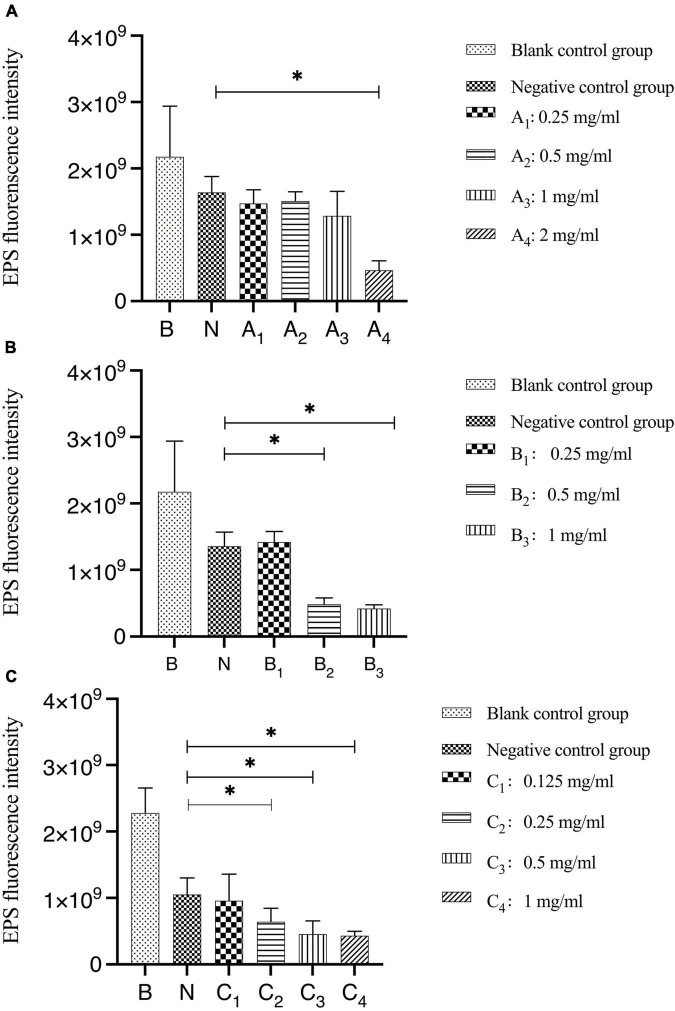
Quantitative analysis of the total amount of EPS in biofilms after bovine trypsin treatment for 30 s, 1 min, and 3 min. **(A–C)** Are the quantitative analysis of the total amount of EPS in the biofilms after bovine trypsin treatment for 30 s, 1 min, and 3 min, respectively. *Indicates that there is a statistically significant difference compared with the negative control group (*P* < 0.05).

The results of the quantitative analysis of biofilm EPS after bovine trypsin treatment for 30 s, 1 min, and 3 min are shown in Figure 4. In Figure 4A, the difference between the negative control group and group A_4_ was statistically significant (*P* < 0.05); in Figure 4B, the negative control group was significantly different from groups B_2_ and B_3_, respectively (*P* < 0.05); in Figure 4C, there were significant differences between the negative control group and groups C_2_, C_3_, and C_4_, respectively (*P* < 0.05). The results showed that when bovine trypsin acted for 30 s, 1 min, and 3 min, the minimum effective concentrations of EPS were 2, 0.5, and 0.25 mg/ml, respectively.

### Morphological changes and quantitative analysis of live and dead bacteria after bovine trypsin treatment according to confocal laser scanning microscopy observation

**FIGURE 5:**
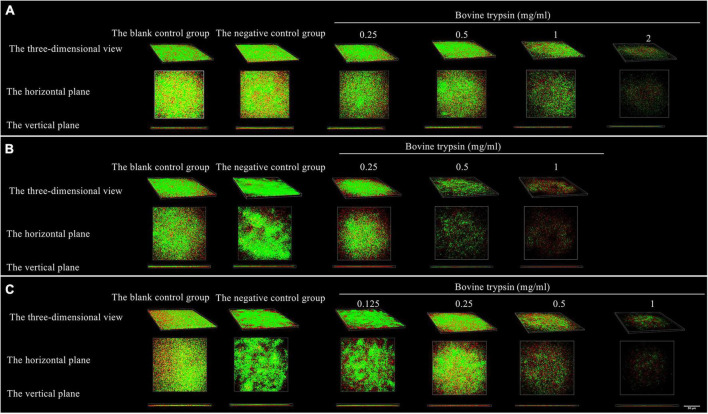
The three-dimensional morphology observation of live and dead bacteria after 30 s, 1 min, and 3 min of bovine trypsin treatment by CLSM. **(A–C)** Are the form of live and dead bacteria in the biofilm after 30 s, 1 min, and 3 min of ovine trypsin treatment, respectively.

Figure 5 shows the morphology of live and dead bacteria after the action of bovine trypsin on the biofilm for 30 s, 1 min, and 3 min. The live bacteria show green fluorescence, and the dead bacteria show red fluorescence. After the action of bovine trypsin, as the concentration increased, the action time increased, the biofilm structure became dispersed, and the green and red fluorescence decreased.

**FIGURE 6:**
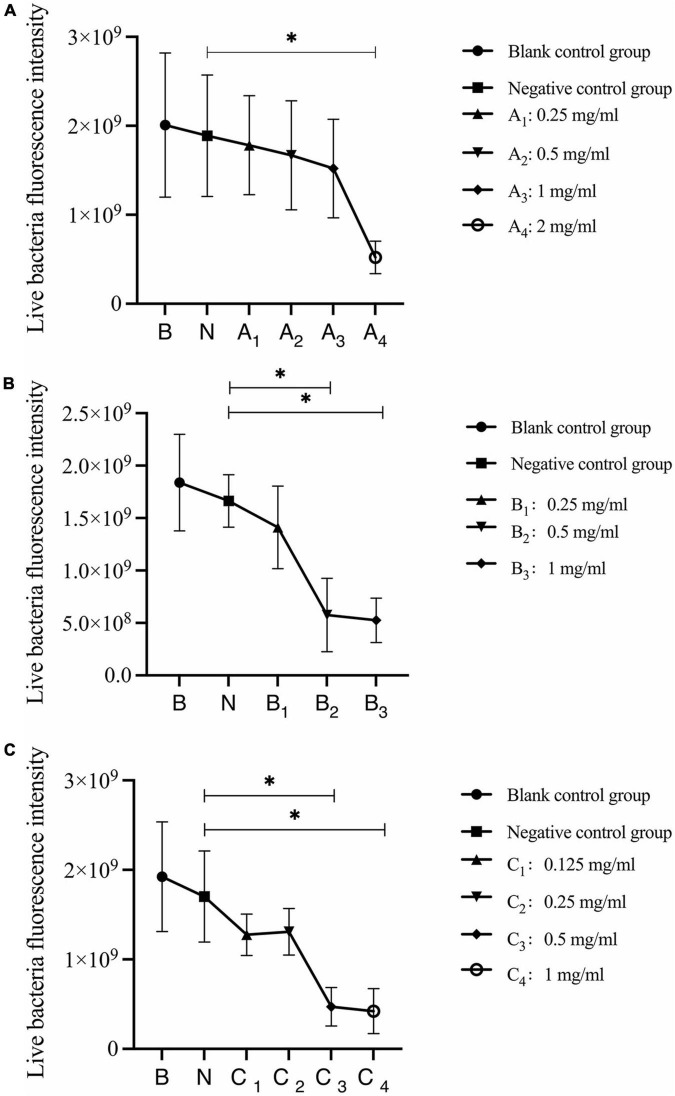
Quantitative analysis of live bacteria in biofilms after bovine trypsin treatment for 30 s, 1 min, and 3 min. **(A–C)** Show the quantitative analysis of biofilm live bacteria after 30 s, 1 min, and 3 min of bovine trypsin treatment, respectively. *Indicates that there is a statistically significant difference compared with the negative control group (*P* < 0.05).

Quantitative analysis of live bacteria after 30 s, 1 min, and 3 min of bovine trypsin action on the biofilms are shown in Figure 6. In Figure 6A, the difference between the negative control group and group A_4_ was statistically significant (*P* < 0.05); in Figure 6B, the negative control group was compared with groups B_2_ and B_3_, and the difference was statistically significant (*P* < 0.05); in Figure 6C, the negative control group was compared with groups C_3_ and C_4_, and the difference was statistically significant (*P* < 0.05). The results suggested that when bovine trypsin acted for 30 s, 1 min, and 3 min, the lowest effective concentrations for reducing live bacteria in the biofilm were 2, 0.5, and 0.5 mg/ml, respectively.

**FIGURE 7:**
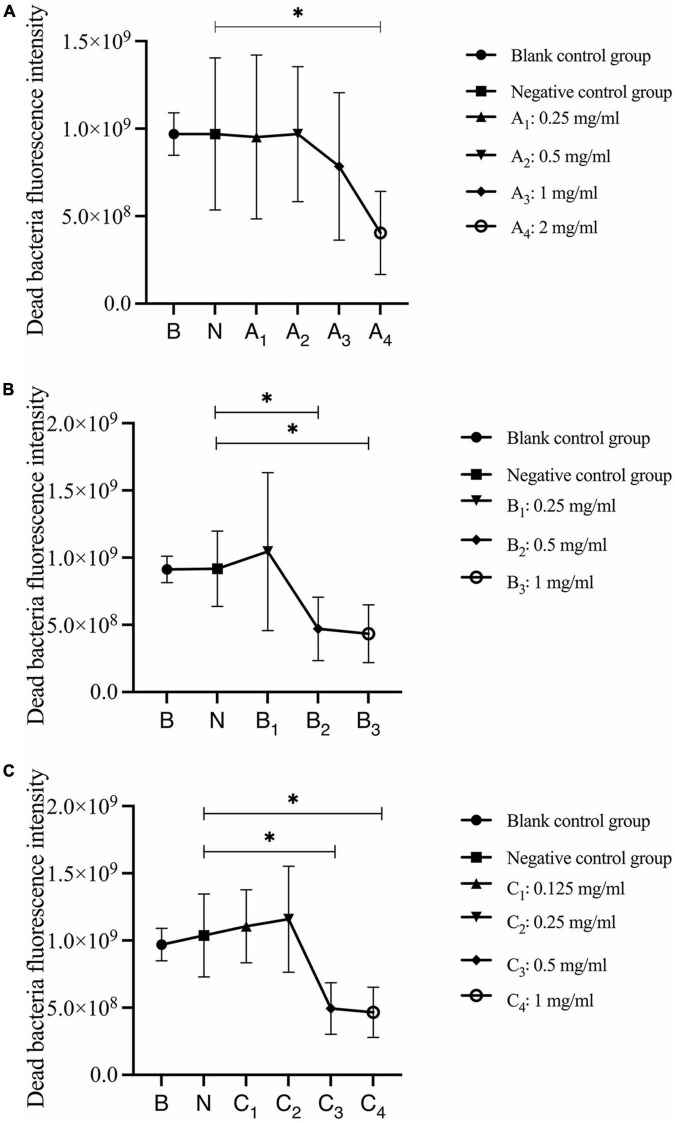
Quantitative analysis of dead bacteria in biofilm after bovine trypsin treatment for 30 s, 1 min, and 3 min. **(A–C)** Show the quantitative analysis of biofilm dead bacteria after 30 s, 1 min, and 3 min of bovine trypsin treatment, respectively. *Indicates that there is a statistically significant difference compared with the negative control group (*P* < 0.05).

Quantitative analysis of dead bacteria after 30 s, 1 min, and 3 min of bovine trypsin acting on biofilms are shown in Figure 7. In Figure 7A, the negative control group was compared with group A4, and the difference was statistically significant (*P* < 0.05); in Figure 7B, the negative control group was compared with groups B_2_ and B_3_, and the difference was statistically significant (*P* < 0.05); in Figure 7C, the negative control group was compared with groups C_3_ and C_4_, and the difference was statistically significant (*P* < 0.05). The results suggested that when bovine trypsin acted for 30 s, 1 min, and 3 min, the lowest effective concentrations for reducing dead bacteria in the biofilm were 2, 0.5, and 0.5 mg/ml, respectively.

**FIGURE 8:**
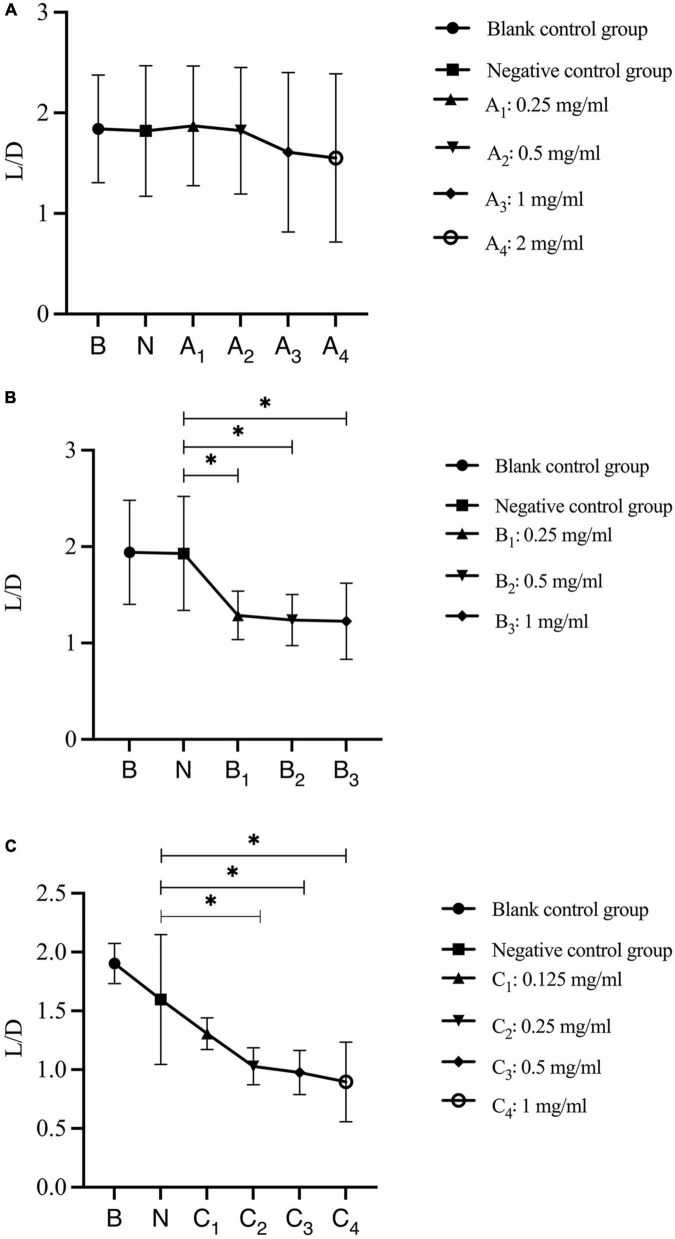
Changes in the ratio of live/dead bacteria after bovine trypsin treatment for 30 s, 1 min, and 3 min. **(A–C)** Show the quantitative analysis of the ratio of live bacteria/dead bacteria in the biofilms after 30 s, 1 min, and 3 min of bovine trypsin treatment, respectively. *Indicates that there is a statistically significant difference compared with the negative control group (*P* < 0.05).

The analysis of changes in the ratio of live bacteria to dead bacteria after 30 s, 1 min, and 3 min of bovine trypsin action on the biofilm are shown in Figure 8, respectively. In Figure 8A, there was no significant difference between the negative control group and the bovine trypsin treatment groups (*P* > 0.05); in Figure 8B, the negative control group was compared with groups B_1_, B_2_, and B_3_, and the difference was statistically significant (*P* < 0.05); in Figure 8C, the negative control group was compared with groups C_2_, C_3_, and C_4_, and the difference was statistically significant (*P* < 0.05). The results showed that when bovine trypsin acted for 30 s, all concentrations of bovine trypsin did not significantly change the ratio of live/dead bacteria. Moreover, when bovine trypsin acted for 1 min and 3 min, the lowest effective concentration for reducing the ratio of live/dead bacteria was 0.25 mg/ml.

## Discussion

The main purpose of periodontal therapy is to eliminate infection, which can be achieved with mechanical debridement and antimicrobial agents. However, bacteria in periodontal pockets often exist in the form of biofilms, which can make them more resistant to antimicrobials. In addition, the complex anatomical structure of the root and periodontal system provides a hiding place for the biofilm, which makes it difficult to remove the biofilm in these parts with traditional mechanical debridement. Moreover, due to the protection offered by EPS, the effect of antibacterial drugs on the biofilm is not strong enough. Therefore, the chemical drugs used to remove the biofilm in the periodontal pocket should also possess the ability to destroy the biofilm EPS. EPS can be used as a target to destroy the structure and stability of the biofilm, promote the degradation of the biofilm and cause the separation of bacteria in it, and sensitize the bacteria in the biofilm. Meanwhile, it can be combined with antibacterial drugs to enhance the effect of antibacterial drugs (Wolfmeier et al., 2018; Zhao et al., 2018). However, the specific target proteins of bovine trypsin to degrade periodontitis-related microbial multispecies biofilms have not been fully studied. The mechanism by which bovine trypsin degrades biofilms has been somewhat understood in recent years, but many details remain unclear, and more studies are needed to fill in the gaps.

The results of this study showed that after the action of bovine trypsin, the biofilm degraded, EPS dispersed and the total amount decreased; thus, bovine trypsin could degrade EPS. The reduction of EPS by bovine trypsin was positively correlated with the duration of action and the concentration of bovine trypsin. With the prolongation of bovine trypsin action time, EPS decreased more obviously, and the minimum effective concentration decreased. When the minimum effective concentration was reached, the total amount of EPS decreased significantly. According to previous researches, the main reports on the biofilm degradation mechanism mainly include the degradation of EPS, which is related to the hydrolysis of composition-related proteins of EPS. It is speculated that the mechanism of reduction of EPS is related to the composition-related proteins of EPS that can be hydrolyzed by bovine trypsin: (1) Bovine trypsin hydrolyzes bacterial cell surface proteins. The EPS protein components of the biofilms of bacteria such as *Staphylococcus aureus* include cell surface proteins, mainly composed of fibronectin binding protein A (FnBPA), fibronectin binding protein B (FnBPB), Biofilm associated protein (Bap), Clustering factor B (ClfB) (Lister and Horswill, 2014). The extracellular serine protease (Esp) secreted by *Staphylococcus epidermidis* hydrolyzes Bap associated with the formation of *Staphylococcus aureus* biofilms (Sugimoto et al., 2013). Bovine trypsin also acts as a serine protease; thus, it is speculated that the reduction of EPS in the biofilm of periodontitis-related microorganisms established in this experiment may be related to the hydrolysis of cell surface proteins; (2) Bovine trypsin can hydrolyze bacterial extracellular proteins, which are an important part of EPS. Protease can lead to the hydrolysis of proteins in EPS and reduce the stability of biofilm and is the most potent biofilm-degrading enzyme (Lister and Horswill, 2014). Proteinase K, trypsin, and dispase B can all hydrolyze protein components in the EPS of *staphylococcal* biofilms, degrade biofilms, and promote the dispersion of established biofilm colonies (Chaignon et al., 2007). It is speculated that trypsin acts on the biofilm in this experiment, which may be related to the hydrolysis of extracellular proteins.

Bacterial biomasses are reduced by the action of bovine trypsin, and the ratio of live/dead bacteria is negatively correlated with the action time and concentration of bovine trypsin. When the action time is short, after the bovine trypsin concentration reaches the minimum effective concentration, the biofilm structure is effectively destroyed, the biofilm is dispersed, and the live and dead bacteria are reduced. It is speculated that the reasons are as follows: (1) EPS is hydrolyzed, causing the bacteria embedded in the biofilm EPS to detach from the biofilm; (2) Bovine trypsin destroys the adhesion of the bacteria, and the bacteria detach from the biofilm. When the action time is prolonged, before the bovine trypsin concentration reaches the minimum effective concentration that reduces the total amount of live and dead bacteria, the EPS on the surface of the biofilm is hydrolyzed, the bacteria of the biofilm is separated from the biofilm partially, and there are more live bacteria at this site, resulting in a decrease in live bacteria. After the EPS surface part was hydrolyzed, the bacteria without EPS protection that contacted bovine trypsin died, and the dead bacteria in the middle and lower layers of the biofilm remained in the biofilm; therefore, the amount of dead bacteria increased, which led to a decrease in the ratio of live bacteria/dead bacteria. After the bovine trypsin concentration reaches the minimum effective concentration to reduce the total amount of live and dead bacteria, the biofilm structure is destroyed completely, the biofilm is dispersed, and the bacteria are separated from the biofilm; therefore, the total amount of bacteria reduces. After the biofilm is dispersed, the bottom layer persists, the bacteria partially adheres to the surface of the glass slide, and the number of dead bacteria in the biofilm gradually increases from the surface layer to the bottom layer; therefore, the ratio of live bacteria/dead bacteria decreases.

After the action of bovine trypsin, the bacteria in the biofilm became dispersed and the bacteria died, but the bacterial morphology did not change significantly. Bacterial death can be achieved in two way: (1) destruction of the cell membrane, resulting in cell death and deformation of the bacteria; and (2) targeted binding to the DNA gyrase/topoisomerase IV-DNA complex, preventing DNA replication and breaking it, which results in bacterial death and may not affect the bacterial morphology in a short time (Hooper and Jacoby, 2016). There was no obvious change in bacterial morphology in this experiment, and the specific mechanism needs to be further studied.

Further research on this topic will lead to the development of new therapeutic options for biofilm-mediated periodontitis.

## Conclusion

Periodontitis-related microbial multispecies biofilm established in this study is formed by the EPS encapsulating the bacterial group and has a three-dimensional network structure, which is in line with the characteristics of biofilms. Bovine trypsin can destroy the biofilm structure, disperse the biofilm and bacteria, and significantly reduce the EPS and bacterial biomass. The effect of bovine trypsin on degrading periodontitis-related microbial multi-species biofilm was positively correlated with the action time and concentration.

## Data availability statement

The raw data supporting the conclusions of this article will be made available by the authors, without undue reservation.

## Author contributions

JZ contributed to data acquisition, data analysis, and manuscript preparation. XM, QH, YH, and YL contributed to data acquisition and data analysis. LH contributed to the design of the study and professionally revised the manuscript. All authors read and approved the final manuscript.
